# RNA‐seq and ATAC‐seq analysis of CD163 ^+^ macrophage‐induced progestin‐insensitive endometrial cancer cells

**DOI:** 10.1002/cam4.5396

**Published:** 2022-11-14

**Authors:** Lulu Wang, Qiaoying Lv, Pengfei Wu, Shuhan Luo, Sijia Liu, Xiaojun Chen, Xuezhen Luo

**Affiliations:** ^1^ Obstetrics and Gynecology Hospital Fudan University Shanghai China; ^2^ Shanghai Key Laboratory of Female Reproductive Endocrine Related Diseases Obstetrics and Gynecology Hospital Fudan University Shanghai China

**Keywords:** CD163^+^ macrophages, endometrial cancer, extracellular matrix, progesterone receptor, progestin insensitivity

## Abstract

**Background:**

Progestins are used as fertility‐sparing regimens for young patients with stage 1A endometrioid endometrial cancer (EEC) and atypical endometrial hyperplasia (AEH). CD163^+^ macrophages promote estrogen‐dependent EEC development, but whether they induce progestin insensitivity remains unclear. This study aimed to investigate the possible effects of CD163^+^ macrophages on progestin response in AEH/EEC patients.

**Methods:**

The number of infiltrating CD163^+^ macrophages in progestin‐insensitive and ‐sensitive endometrial lesions was compared. The effects of CD163^+^ macrophages on progestin responses and progesterone receptor (PR) expression in EC cells were evaluated in vitro. ATAC‐seq and RNA‐seq were combined to identify molecular/biological changes induced by CD163^+^ macrophages in progestin‐insensitive EC cells.

**Results:**

Increased CD163^+^ macrophage infiltration was significantly associated with progestin insensitivity and longer treatment durations in AEH/EEC patients. Additionally, the number of CD163^+^ macrophages was negatively correlated with PR expression in AEH/EEC tissues. Furthermore, the CD163^+^ macrophage‐mediated microenvironment and secreted cytokines downregulated PR expression and impaired the response of EC cells to medroxyprogesterone acetate (MPA). RNA‐seq analysis demonstrated that CD163^+^ macrophages antagonized PR signaling by blocking or even reversing MPA‐regulated differential gene expression. Based on RNA‐seq and ATAC‐seq analyses, extracellular matrix (ECM) signaling and ECM‐related transcription factors, FOXF2, POU1F1, and RUNX1were identified to potentially be involved in CD163^+^ macrophage‐induced progestin insensitivity in endometrial cancer patients.

**Conclusions:**

We identified CD163^+^ macrophages as an important mediator of progestin desensitization and an unfavorable factor for the efficacy of fertility‐preserving treatment in AEH/EEC patients.

## Graphical abstract

Infiltrating CD163^+^ macrophages were correlated with progestin insensitivity in endometrial cancer. CD163^+^ macrophages antagonize PR signaling, driving EC cell unresponsiveness to progestins. An ECM‐related mechanism might be involved in CD163^+^ macrophage‐induced progestin insensitivity.

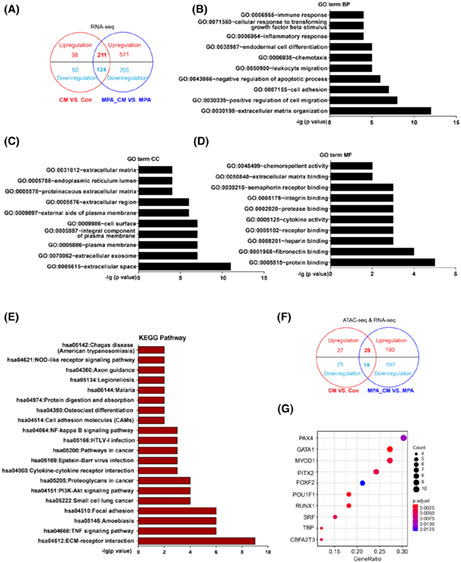

## 1 INTRODUCTION

Endometrial carcinoma (EC) is one of the most common gynecologic malignancies, and its incidence is increasing in younger patients. Epidemiological evidence revealed that approximately 7.1% of EC patients were between 20 and 44 years old at the time of diagnosis, and 70%–88% of them had not completed childbearing.^1^ Because the occurrence of EC is strongly related to prolonged estrogen exposure without progestin protection, synthetic progestins have been administered clinically as fertility‐sparing agents for young patients with atypical endometrial hyperplasia (AEH) and early endometrioid cancer (EEC). Complete response (CR) rates for progestin therapy in EEC patients range from 72.9% to 95.3%, depending on the drugs and regimens used.^2^ However, approximately 30% of EEC patients fail to respond to progestins or exhibit a temporary or partial response.^3^, ^4^ Moreover, the molecular mechanisms underlying progestin insensitivity are poorly understood.

The progesterone receptor (PR) is the primary target of progestins, and the efficacy of progestin treatment in endometrial cancer is mainly mediated by the PR signaling pathway.^5^ It has been widely reported that progestin insensitivity has a strong relationship with the loss of expression, downregulation, or dysfunction of PR.^6^ Therefore, identifying environmental factors that affect the protein expression of PR and desensitize EC cells to progestin is important.

Macrophages, an important component of chronic inflammation, are classified into the pro‐inflammatory M1 phenotype and the anti‐inflammatory M2 phenotype. A previous study reported that the obesity‐related tumor microenvironment recruited macrophages and promoted M2 polarization through the COX‐2/PEG2 pathway in prostate cancer.^7^ Similarly, several studies demonstrated increased CD163^+^ macrophage infiltration with higher pathological grades in abnormal endometrial hyperplasic lesions. We previously demonstrated that cytokines secreted by CD163^+^ macrophages (M2‐like macrophages) induced ERα expression through epigenetic modulation of the ESR1 gene promoter and stabilized ERα through de‐ubiquitination of ERα protein.^8^, ^9^ However, whether CD163^+^ macrophages induce progestin insensitivity remains unclear.

In the present study, we compared the number of infiltrating CD163^+^ macrophages in progestin‐insensitive and progestin‐sensitive AEH/EC patients. In addition, the regulatory effects of CD163^+^ macrophages on progestin sensitivity and PR expression in EC cells were investigated. Furthermore, ATAC‐seq and RNA‐seq analyses were integrated to identify molecular/biological changes induced by CD163^+^ macrophages in progestin‐insensitive EC cells. Our study demonstrated reduced sensitivity of EC cells to progestin treatment in the CD163^+^ macrophage‐induced chronic inflammatory state. CD163^+^ macrophages and cytokines, such as IL10 and TGFβ, downregulated PR protein expression. RNA‐seq analysis revealed that the CD163^+^ macrophage environment antagonized PR signaling via blocking or even reversing MPA‐regulated differential gene expression. By integrating ATAC‐seq and RNA‐seq analyses, extracellular matrix‐related signaling was identified as an important mechanism potentially involved in CD163^+^ macrophage‐induced progestin insensitivity in EC cells.

## 2 MATERIALS AND METHODS

### 2.1 Ethics statement

This study complied with the principles of the Declaration of Helsinki and was approved by the Medical Ethics Committee of the Obstetrics and Gynecology Hospital of Fudan University (approval No.2021–131). All patients signed an informed consent form before participating in the study.

### 2.2 Patients and specimens

A total of 22 endometrial tissues were collected from AEH or EEC patients who received fertility‐preserving treatment at the Obstetrics and Gynecology Hospital of Fudan University between January 2017 and August 2019. All patients were pathologically diagnosed by endometrial biopsy through dilation and curettage under a hysteroscope. Pathologic diagnosis was independently confirmed by two experienced gynecological pathologists, according to the World Health Organization pathological classification (2014). If their opinions differed, a seminar was held in the pathology department to determine the final diagnosis.

The inclusion and exclusion criteria for fertility‐sparing treatment followed National Comprehensive Cancer Network guidelines.^10^, ^11^ The inclusion criteria were as follows: (1) histologically‐proven AEH or well‐differentiated EEC G1 without myometrial invasion; (2) no signs of suspicious extrauterine involvement on enhanced magnetic resonance imaging, enhanced computed tomography or ultrasound; (3) patients younger than 45 years old; (4) strong willingness to preserve fertility; (5) no contraindications for progestin treatment or pregnancy; (6) not pregnant; and (7) good compliance for treatment. Written informed consent was obtained from all patients before initiating treatment. The exclusion criteria were as follows: (1) use of local or systematic progestins with more than 1 month before hysteroscopic evaluation; (2) recurrent AEH or EEC; (3) evidence of myometrial invasion; and (4) loss of follow‐up. All endometrial tissues in this study were obtained before fertility‐preserving treatment or within 1 month of treatment.

We defined progestin insensitivity as meeting one of the following criteria^11^: (1) presented progressive disease at any time during conservative treatment, (2) maintained stable disease after 7 months of first‐line treatment, or (3) did not achieve CR after 10 months of first‐line treatment. The conditions above indicated progestin insensitive (PIS), whereas other conditions were considered progestin sensitive (PS). The clinical characteristics of all enrolled patients are shown in Table 1.

**TABLE 1:** General characteristics of the study population

Variables	Total	Sensitivity	Insensitivity	*p*‐value*
No. of patients	22	13	9	—
Age at diagnosis (year)	30.5 (20–39)	31 (23–39)	29 (20–34)	0.158
BMI (kg/m^2^)	28.08 (20.07–45.17)	28.13 (20.07–45.17)	27.82 (22.68–30.47)	0.815
HOMA‐IR index	3.17 (0.19–22.8)	3.23 (0.19–22.8)	2.93 (1.56–10.51)	0.717
MS	10	6	4	1.000
Hypertension	5	3	2	1.000
Diabetes mellitus	1	0	1	0.409
Nulliparous	17	10	7	1.000
Histologyat diagnosis				1.000
AEH	8	5	3	—
EEC	14	8	6	—
Progestin therapy				0.423
MA	7	4	3	—
MA + MET	6	5	1	—
LNG‐IUS	3	2	1	—
MA + LNG‐IUS	6	2	4	—

*Note*: Data are shown as number or median (range). *p*‐value: comparison between sensitivity and insensitivity group.

Abbreviations: AEH, atypical endometrial hyperplasia; BMI, body mass index; EEC, endometrioid endometrial cancer; HOMA‐IR index, homeostasis model assessment‐insulin resistance index; LNG‐IUS, levonorgestrel intrauterine system; MA, megestrol acetate; MET, metformin; MS, metabolic syndrome.

* All continuous variables were analyzed by Wilcoxon rank sum test. Comparison of distribution of different progestin therapies was analyzed by Fisher's exact test. Comparison of distributions of other categorical variables were all analyzed by Pearson's chi‐square test.

### 2.3 Cell lines and cell culture

The human EC cell line Ishikawa was kindly provided by Dr. Yu Yinhua (MD Anderson Cancer Center), and the human EC cell line ECC‐1 was purchased from American Type Culture Collection (Manassas, VA, USA). The human monocyte cell line (THP‐1) was kindly provided by the Stem Cell Bank, Chinese Academy of Sciences. All cell lines were authenticated by short tandem repeat profiling and were routinely tested for no mycoplasma contamination. ECC‐1 and THP‐1 cells were cultured in RPMI 1640 medium, and Ishikawa cells were cultured in DMEM/F12 medium, both supplemented with 10% fetal bovine serum (FBS), 1% penicillin, and streptomycin. All cells were maintained at 37°C in a humidified incubator containing 5% CO2.

### 2.4 Preparation of human peripheral blood monocyte‐derived macrophages

First, peripheral blood mononuclear cells (PBMCs) were isolated from the buffy coats of healthy donors by density gradient centrifugation with lymphocyte isolation solution (Serumwerk Bernburg AG). Then, CD14^+^ cells were isolated from mononuclear cells by CD14 immunomagnetic beads through positive magnetic selection (Miltenyi Biotec). CD14^+^ monocytes were cultured in RPMI 1640 with 10% FBS and in 6‐well flat‐bottom culture plates at 5 × 10^5^ cells/mL.

### 2.5 Drug intervention

The following drugs were used for cell interventions as indicated: phorbol‐12‐myristate‐13‐acetate (PMA) (Sigma Aldrich, 793,416), recombinant human IL4 (Sigma Aldrich, srp3093), recombinant human IL13 (Sigma Aldrich, srp3274), recombinant human IL10 (Novoprotein, CX04), recombinant human TGFβ (Novoprotein, CA59), recombinant human IL10 neutralizing antibody (Biolegend, 501,427), recombinant human TGFβ neutralizing antibody (Biolegend, 947,303), and medroxyprogesterone acetate (MPA) (Selleck, NSC‐26386). Vehicles included dimethyl sulfoxide (DMSO), phosphate buffer saline (PBS), or PBS supplemented with 0.1% BSA.

THP‐1 cells and PBMCs were first treated with 100 ng/mL PMA for 24 h, respectively, to generate M0 macrophages, which are differentiated but unpolarized macrophages. Then, M0 cells were treated with IL4 (20 ng/mL)/IL13 (20 ng/mL) for 48 h to induce CD163^+^ macrophage polarization. The cell culture supernatant was collected as conditioned medium (CM) after culturing THP‐1 or PBMC‐derived CD163^+^ macrophages for 24 h and used in further studies as indicated. Prior to MPA or CM treatment, cells were cultured in phenol red‐free medium supplemented with 10% charcoal‐stripped FBS.

### 2.6 Enzyme‐linked immunosorbent assay (ELISA)

The levels of IL10 and TGFβ in cell culture supernatants of THP‐1 cells and CD163^+^ macrophages were measured by an ELISA kit (R&D Systems) according to the manufacturer's instructions.

### 2.7 Flow cytometry

Macrophages were incubated with fluorescent‐tagged antibodies for flow cytometry as follows: anti‐Human CD163‐PE, anti‐Human IL10‐APC, and anti‐Human TGFβ‐BV421 (all Biolegend, USA). Then, macrophages were detected by flow cytometry (Beckman). ECC‐1 and Ishikawa cells (300,000 cells/well) were seeded in 6‐well plates and allowed to adhere to culture plates overnight. Cells were then treated with MPA, CM, and IL10/TGFβ for 48 h. The cells were then trypsinized with EDTA‐free trypsin and washed twice with cold PBS. The cells were re‐suspended in 100 μl of binding buffer and stained with 1 μl of annexin V‐FITC and 1 μl of PI working solution for 15 min at room temperature in the dark (Dojindo, Japan). Finally, the cell apoptotic level was determined using a flow cytometer (Beckman). As negative controls, isotype‐matched antibodies with corresponding fluorescent labels were used.

### 2.8 Real‐time quantitative PCR (RT‐qPCR)

Total RNAs were extracted using an RNA Purification Kit (EZBioscience, B0004). After removing genomic DNAs with a DNA remover, total RNAs were reverse transcribed into cDNAs using the Reverse Transcription Kit (EZBioscience, A0010GQ). cDNA amplification was performed using the TB Green® Premix Ex Taq™ II (TaKaRa, RR820A). The 2^−ΔΔCt^ method was used to calculate gene expression levels relative to GAPDH. Primers were listed in Table S1.

### 2.9 Western blotting analysis

Western blotting analysis was conducted as previously described.^12^ The following primary antibodies were used: PR (Santa Cruz Biotechnology, sc‐166,169), cyclin D1 (Cell Signaling Technology, E3P5S), p21 (Cell Signaling Technology, 12D1), p27 (Cell Signaling Technology, D69C12), and β‐actin (Huabio, M1210).

### 2.10 Cell viability assay

ECC‐1 and Ishikawa cells (3000 cells/well) were seeded in 96‐well plates, allowed to adhere overnight, and treated with MPA, CM, or cytokines for 48 h. Cell viability was detected using Cell Counting Kit‐8 (CCK‐8) in accordance with the manufacturer's instructions (DOJINDO, Japan).

### 2.11 Immunohistochemical (IHC) staining

IHC staining was performed as previously described.^13^ Primary antibodies used in IHC staining included CD163 (Abcam, ab182422) and PR (Abcam, ab32085). Semi‐quantitative optical analysis was performed as previously described.^14^

### 2.12 ATAC‐seq and RNA‐Seq analyses

Ishikawa cells were harvested from 6‐cm dishes in 1 ml Trizol (Invitrogen, Carlsbad, CA, USA) in accordance with the manual after a 24 h induction with 20 μM MPA or DMSO in the presence or absence of CM. RNAs were subjected to RNA‐Seq analysis with a BGISEQ‐500 system by Beijing Institute (BGI), China. RNA integrity was examined using a NanoDrop spectrophotometer (Thermo Fisher) and Bioanalyzer 2100 (Agilent). RNAs were sheared and reverse transcribed into cDNAs using random primers for library construction. Subsequently, sequencing was performed using the prepared library.^15^ All generated raw sequencing reads were filtered to obtain clean reads stored in the FASTQ format. Bowtie2 and HISAT were applied to align the clean reads to the reference gene and genome, respectively.^16^, ^17^ The expression level (FPKM) of genes was calculated by RSEM.^18^ Read counts for each gene were determined using the SubRead package.^19^ Normalization and differential expression analysis were performed using DeSeq2.^20^

To investigate chromatin accessibility, Ishikawa cells in 10‐cm dishes were collected after 24 h of treatment with MPA or DMSO in the presence or absence of CM. ATAC‐seq was performed as described previously.^21^ Libraries were pooled in equimolar ratios with barcodes and sequenced on the BGISEQ‐500 platform (BGI‐Shenzhen, China).

For RNA‐seq analysis, genes with |Log2 Fold change| > 0.5849 and adjusted *p* < 0.05 were selected as differentially expressed genes (DEGs). For ATAC‐seq analysis, opening or closing peaks with |Log2 Fold change| > 0.5849 and non‐adjusted *p* < 0.05 were selected according to MAnorm analysis. Raw and processed data are available from the corresponding author on reasonable request.

### 2.13 Bioinformatic analysis

To investigate the biological relationship between PGR (PR coding gene) and M1/M2‐like macrophage‐secreted cytokine genes, Ingenuity Pathway Analysis software (IPA, Ingenuity System; http://www.ingenuity.com) was used for the analysis of functional biological networks. Network analysis was performed by uploading gene IDs into IPA, carefully ensuring that each gene was uniquely and accurately recognized by the software. Gene interaction networks were then generated automatically (i.e., independent of investigators). Pathways and networks were ranked according to the number of molecules with a cutoff value (*p* < 0.05) for significantly enriched pathways/networks involving PGR, and cytokine genes were identified using the “Compare” module in IPA.

The bioinformatics analyses used in integration analysis of ATAC‐Seq and RNA‐Seq included REACTOME pathways, transcription factor (TF) prediction, and Motif enrichment. (1) Based on overlapping DEGs by ATAC‐Seq and RNA‐Seq integrated analysis, top ten REACTOME pathways were enriched, and DEGs in the pathways potentially regulating progestin insensitivity were first screened out; (2) potential TFs that regulate the expression of the overlapping DEGs were enriched by HOMER Software, and DEGs‐encoding TFs with p value <0.05 were screened out; and (3) Motif enrichment was performed to identify important TFs by using homer peak analysis software.

We employed deepTools^22^ to plot the heatmap of the read coverage. Briefly, the sorted bam files were used to calculate the scores per genome regions by *computeMatrix* with scale‐regions mode, and the heatmap was generated based on the score matrix by *plotHeatmap* with default options.

### 2.14 Statistics analysis

Statistical analyses were performed using SPSS statistical software (version 23.0, IBM). All experiments were repeated at least three times. Student's *t*‐test, one‐way or two‐way ANOVA, and Spearman's correlation analysis were used for further statistical analyses. The clinical characteristics of patients were analyzed using the Mann–Whitney U test, chi‐square test, or Fisher's exact test as appropriate. Statistical significance was determined as *p* < 0.05 in two‐sided tests.

## 3 RESULTS

### 3.1 Increased number of infiltrating CD163 ^+^ macrophages in AEH and EEC patients with progestin insensitivity

**FIGURE 1:**
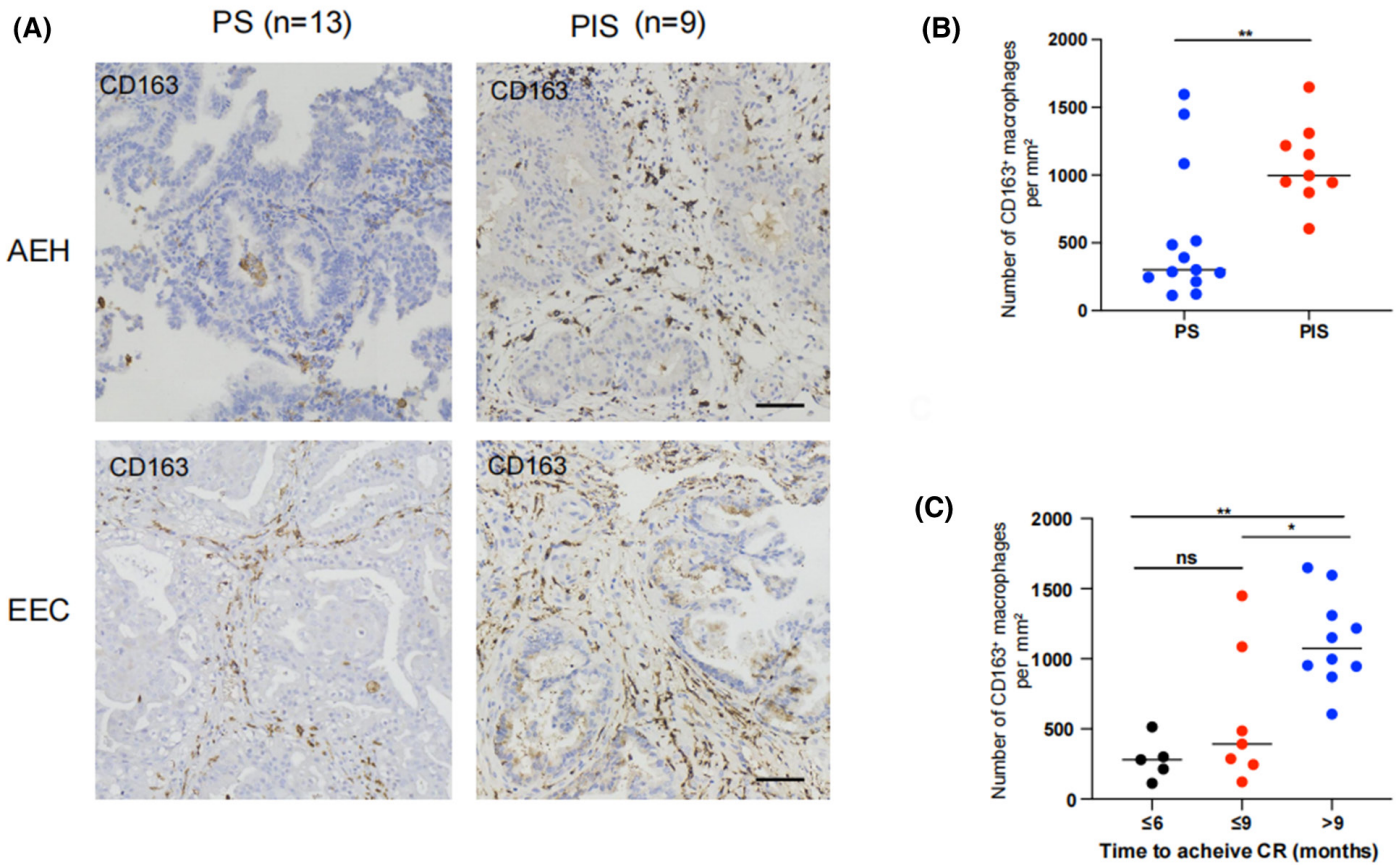
Increased number of infiltrating CD163^+^ macrophages in AEH/EEC tissues of patients with progestin insensitivity. (A) Representative images of IHC staining for CD163 in AEH and EEC tissues before progestin therapy in progestin‐sensitive or progestin‐insensitive groups. Scale bar: 50 μm. (B) The quantification of CD163^+^ macrophages per mm^2^ was analyzed in PS or PIS AEH and EEC tissues. (C) The number of CD163^+^ macrophages per mm^2^ was compared in AEH and EEC tissues according to the time to achieve CR. Statistical analysis was conducted using two‐tailed Student's *t*‐test and ANOVA. PIS, progestin insensitive; PS, progestin sensitive; CR, complete response. ns, not significant; *p < 0.05; **p < 0.01; ***p < 0.001 compared with the control.

To determine whether CD163^+^ macrophages regulate progestin sensitivity in patients with AEH/EEC, we first analyzed the relationship between CD163^+^ macrophage infiltration and progestin sensitivity. CD163 was used to identify M2‐like macrophages in AEH or EEC specimens with PS (*n* = 13) and PIS (*n* = 9). The clinical characteristics of PS and PIS groups were comparable (Table 1). IHC staining analysis revealed that CD163 was mainly expressed in tumor stromal regions in AEH/EEC tissues (Figure 1A). The number of CD163^+^ macrophages in AEH/EEC specimens was counted and analyzed by semi‐quantitative optical analysis. We found that the number of infiltrating CD163^+^ macrophages in the tumor stroma of patients with PIS was significantly higher than that in the PS group (Figure 1B). Furthermore, infiltrating CD163^+^ macrophages increased gradually with the time to achieve CR (Figure 1C). The increased number of infiltrating CD163^+^ macrophages indicated their important roles in mediating progestin insensitivity in AEH/EEC patients.

### 3.2 CD163 ^+^ macrophages decreased the sensitivity of EC cells to progestin therapy

Based on the clinical evidence above, we assumed that infiltrating CD163^+^ macrophages might contribute to progestin sensitivity in AEH/EEC. Accordingly, CD163^+^ macrophages were successfully induced from THP‐1 cells (Figure S1) and PBMCs, respectively (Figure S2B). Macrophages were terminally differentiated after treatment with PMA and IL4/IL13 and showed higher levels of M2 type macrophage markers, including CD163, IL10, and TGFβ. First, we co‐cultured EC cells with different amounts of CD163^+^ macrophage‐derived CM diluted with normal medium (NM). As shown in Figure S2A, cells cultured with diluted CM (CM:NM = 1:2) showed the similar cell viability compared with cells treated with complete NM. Therefore, diluted CM (CM:NM = 1:2) was applied for the following study.

**FIGURE 2:**
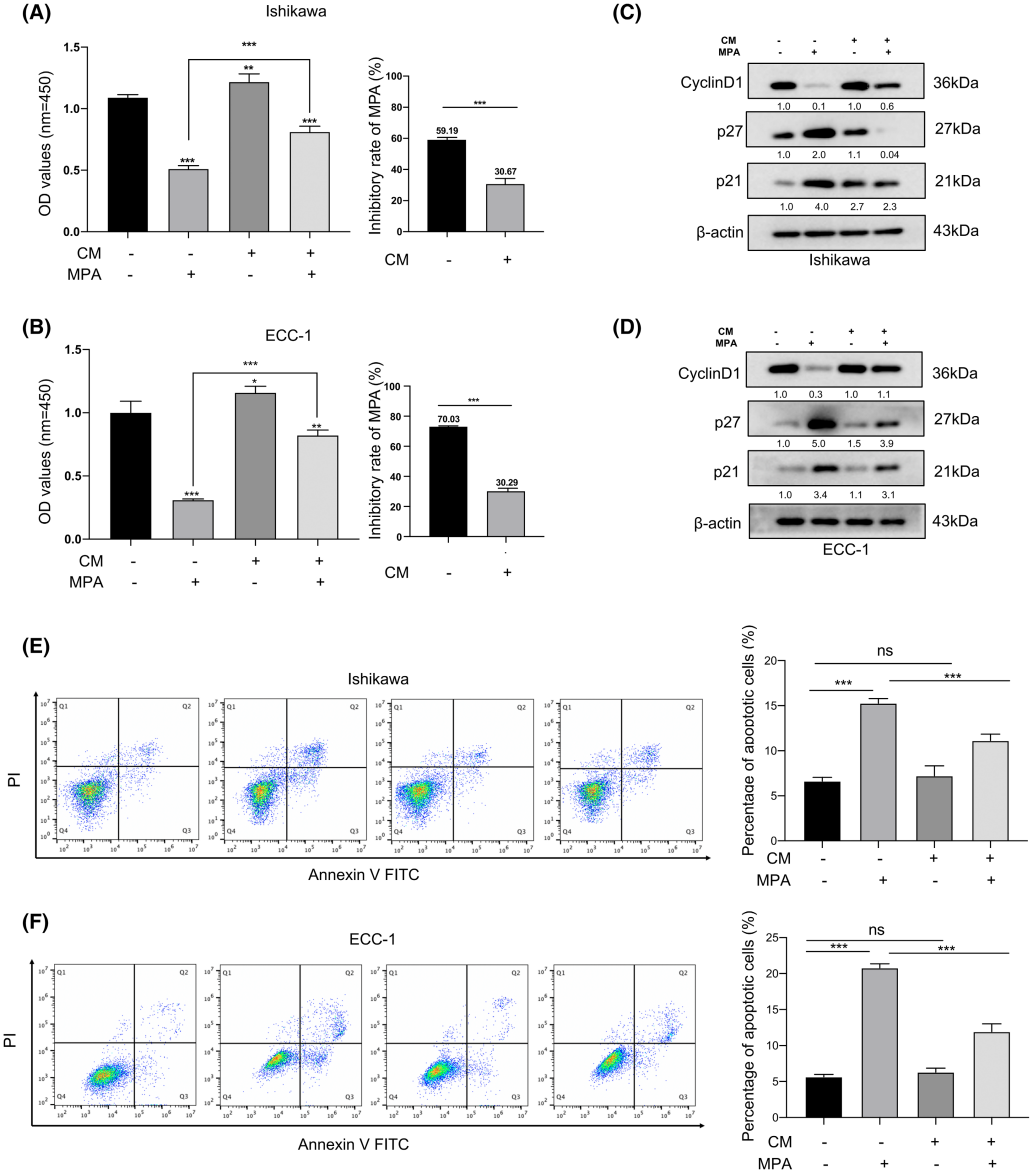
CD163^+^ macrophages decreased the sensitivity of EC cells to progestin therapy. (A, B) CD163^+^ macrophages decreased the MPA‐mediated inhibitory effect on EC cell proliferation. Ishikawa (A) and ECC‐1 (B) cells were cultured together with the indicated dilutions of conditional medium (CM) derived from macrophages and/or MPA for 48 h. Cell viability was evaluated with CCK‐8 test (left), and the inhibitory rate was calculated (right). (C, D) CD163^+^ macrophages inhibited MPA‐mediated downregulation of cyclin D1 protein and upregulation of p21 and p27 protein in the cell cycle pathway. Endometrial cancer cells were treated with CM and/or MPA for 24 h, and the expression of cell cycle proteins was analyzed by Western blotting analysis. (E, F) CD163^+^ macrophages decreased MPA‐induced apoptotic effects on EC cells. EC cells were cultured with CM and/or MPA for 48 h, and the apoptotic rate was determined by flow cytometry. All values are presented as the mean ± SD. ns, not significant; *p < 0.05; **p < 0.01; ***p < 0.001 compared with the control.

We next explored the response of EC cells to MPA in the presence or absence of CM. We found that CM stimulated the proliferation of ECC‐1 and Ishikawa cells, whereas the inhibitory rate of MPA was significantly decreased in Ishikawa cells (59.18 ± 0.7975% vs. 30.67 ± 2.050%, *p* < 0.0001) and ECC‐1 (70.03 ± 0.2963% vs. 30.29 ± 0.9188%, *p* < 0.0001) (Figure 2A,B). PBMC‐derived CD163^+^ macrophages were employed to further confirm our findings. CM from CD163^+^ macrophages could induce a significant decline of the inhibitory rate of MPA in Ishikawa (31.20 ± 0.6673%vs. 57.20 ± 0.2406%, *p* < 0.0001) and ECC‐1 (22.20 ± 1.477% vs. 68.39 ± 0.2782%, *p* < 0.0001) cells compared with CM from M0 macrophages (Figure S2C,D). Progestins induce the cell cycle arrest of EC cells by downregulating cyclin D1 and upregulating p21 and p27 expression.^23^ Cyclin D1 is an essential factor for cell cycle G1/S transition, whereas p21 and p27 bind to cyclin‐CDK complexes and induce cell cycle arrest.^24^ As shown in Figure 2C,D, MPA treatment alone significantly decreased cyclin D1 expression and increased p21 and p27 expression in EC cells. CM of CD163^+^ macrophages generated from THP‐1 cells significantly diminished the MPA‐induced cell cycle inhibition. Consistent results were obtained when EC cells treated with CM of CD163^+^ macrophages generated from PBMCs (Figure S2E,F). Another important indicator of effective progestin treatment is an increase in apoptotic EC cells. We next evaluated whether CM affects progestin‐induced EC cell apoptosis using an annexin‐V and PI assay. Compared with the MPA alone group, the proportion of early and late apoptotic cells was significantly decreased in the MPA and CM combination group of ECC‐1 cells (20.78 ± 0.5485% vs. 11.94 ± 1.075%, *p* < 0.0001) and Ishikawa cells (20.78 ± 0.5485% vs. 11.94 ± 1.075%, *p* < 0.0001) (Figure 2D). Collectively, these results demonstrated that CD163^+^ macrophages decreased the progestin sensitivity of EC.

### 3.3 CD163 ^+^ macrophages and secreted cytokines decreased PR protein in EC cells

**FIGURE 3:**
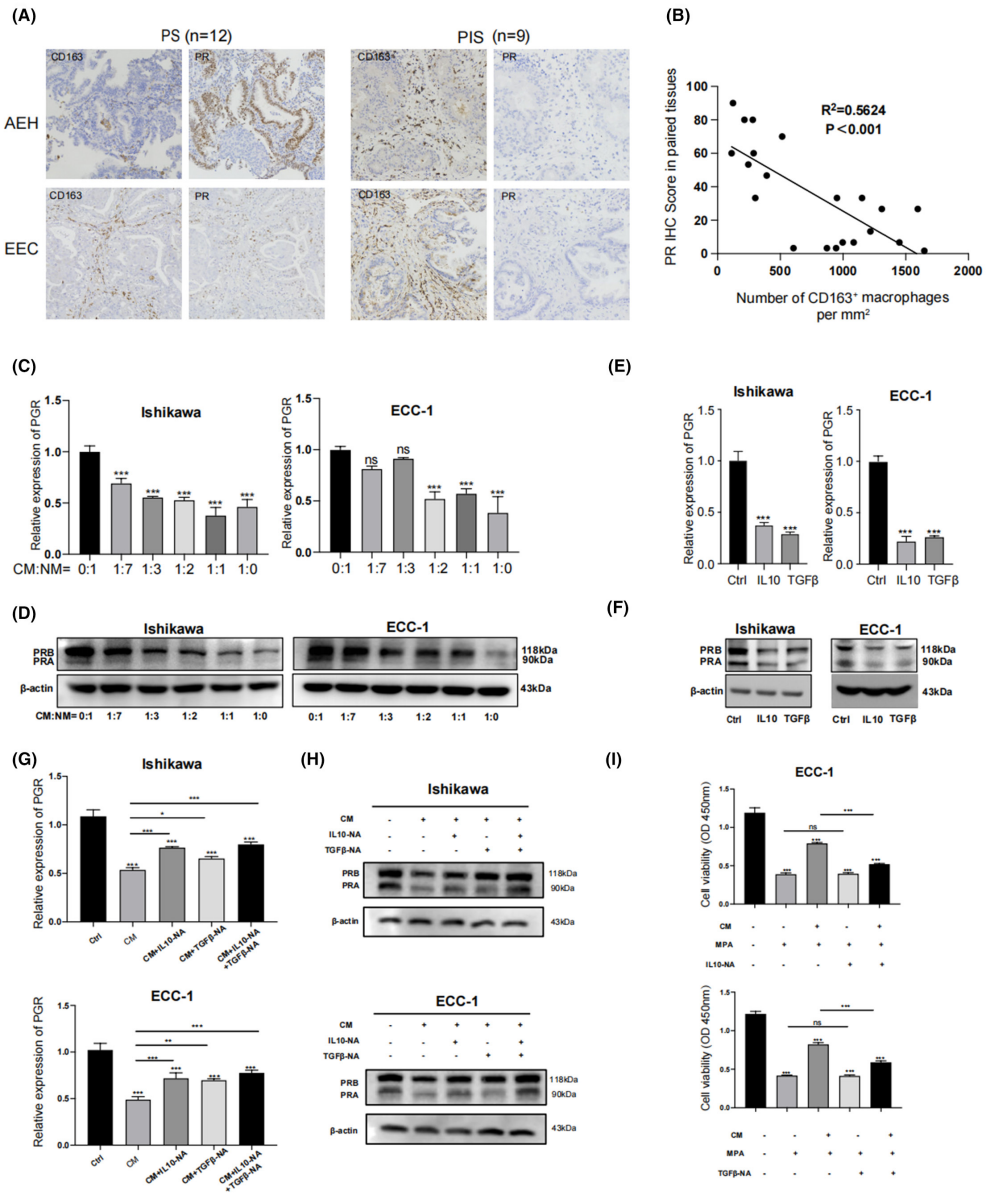
CD163^+^ macrophages and secreted cytokines decreased PR protein in EC cells. (A) Representative IHC images for CD163 (Figure 1A) and PR expression in AEH and EEC samples before progestin therapy in progestin‐sensitive (PS) or progestin‐insensitive (PIS) groups. Scale bar: 50 μm. Sequential tissue slices from each patient in Figure 1A were further immunostained for PR marker, except for one patient in PS group without enough tissue specimens. (B) The staining intensity of PR was scored by semi‐quantitative optical analysis. Correlation between the number of CD163^+^ macrophages per mm^2^ and PR score was analyzed by Spearman's correlation. (C) CM derived from CD163^+^ macrophages inhibited the transcriptional level of PR in endometrial cancer cells in a dose‐dependent manner. Ishikawa and ECC‐1 cells were treated with the indicated dilutions of CM from CD163^+^ macrophages for 24 h. Relative PR mRNA level was analyzed by real‐time PCR. (D) CM derived from CD163^+^ macrophages downregulated the protein expression of PR in EC cells in a dose‐dependent manner. Ishikawa and ECC‐1 cells were treated with the indicated dilution CM from CD163^+^ macrophages for 24 h and then harvested for PR protein analysis by Western blotting. (E) IL10 and TGFβ inhibit the PR transcriptional level in Ishikawa and ECC‐1 cells. Endometrial cancer cells were incubated with IL10 and TGFβ for 24 h and then harvested for analyzing the PR mRNA level by real‐time PCR. (F) IL10 and TGFβ downregulated PR expression in Ishikawa and ECC‐1 cells. Endometrial cancer cells were incubated with IL10 and TGFβ for 48 h and then harvested for analyzing PR protein by Western blotting. (G, H) IL10 and TGFβ neutralizing antibody (NA) antagonized the PR downregulation effects induced by CM from CD163^+^ macrophages. RT‐qPCR and Western blot were used to detect PR mRNA and protein expression levels in EC cells treated with or without CM from CD163^+^ macrophages or IL10/TGFβ‐NA. I. IL10 and TGFβ‐NA reversed the progestin insensitivity induced by CM in ECC‐1 cells. ECC‐1 cells were cultured together with CM and/or MPA in the presence or absence of IL10/TGFβ‐NA for 48 h before the CCK‐8 test. All values are presented as mean ± SD. ns, not significant; *p < 0.05; **p < 0.01; ***p < 0.001 compared with the control.

It is well established that the effects of progestin on the endometrium are mediated by interactions with the PR and PR‐mediated signaling pathway.^25^ We asked whether PR expression was downregulated in CD163^+^ macrophage‐induced PIS EC cells. First, IHC staining of serial endometrial tissue sections showed increased infiltrating CD163^+^ macrophages in the stroma and decreased PR expression levels in adjacent epithelial and stroma regions (Figure 3A). We further analyzed the correlation between the number of CD163^+^ macrophages and epithelial PR expression using Pearson's correlation test, and the results demonstrated that the number of CD163^+^ macrophages was significantly negatively correlated with the PR IHC score (Figure 3B). To determine the PR expression status after treatment with CM from CD163^+^ macrophages, we analyzed the changes in PR expression in EC cells. Real‐time PCR showed that treatment with CM derived from CD163^+^ macrophages decreased the PR mRNA level in a dose‐dependent manner (Figure 3C). Similarly, Western blotting analysis demonstrated that CM from both THP‐1 and PBMC‐derived CD163^+^ macrophages reduced PRA and PRB protein expression in ECC‐1 and Ishikawa cells dose‐dependently (Figure 3D and Figure S2G). This suggested that PR downregulation was an important event for the CD163^+^ macrophage‐induced desensitization of EC cells to progestins.

Our above findings showed that CM derived from CD163^+^ macrophages induced progestin insensitivity and downregulated PR expression. We predicted that cytokines secreted by CD163^+^ macrophages might be involved in the CM‐mediated progestin insensitivity. To test this, IPA was used to determine whether a potential regulating network existed between CD163^+^ macrophage‐related cytokine genes and *PGR*. As shown in Figure S3A, the IPA network illustrated possible molecular interactions among several M1/M2 cytokines and *PGR*, and IL10 and TGFβ were identified as potential cytokines regulating *PGR* in EC cells. ELISA further confirmed that THP‐1‐derived CD163^+^ macrophages exhibited a significantly increased secretion of IL10 and TGFβ than the THP‐1 cells (Figure S2H). Next, IL10 or TGFβ could inhibit *PGR* transcription and PR protein expression in EC cells (Figure 3E,F). Furthermore, CM‐induced PR downregulation effects could be antagonized by IL10/TGFβ neutralizing antibodies in EC cells (Figure 3G,H). IL10/TGFβ neutralizing antibodies could also reverse the progestin insensitivity induced by CM in ECC‐1 cells (Figure 3I).

Taken together, these findings indicate that CD163^+^ macrophages decreased PR expression and desensitized EC cells to progestins.

### 3.4 CD163 ^+^ macrophages antagonized PR signaling, driving EC cell unresponsiveness to progestins

**FIGURE 4:**
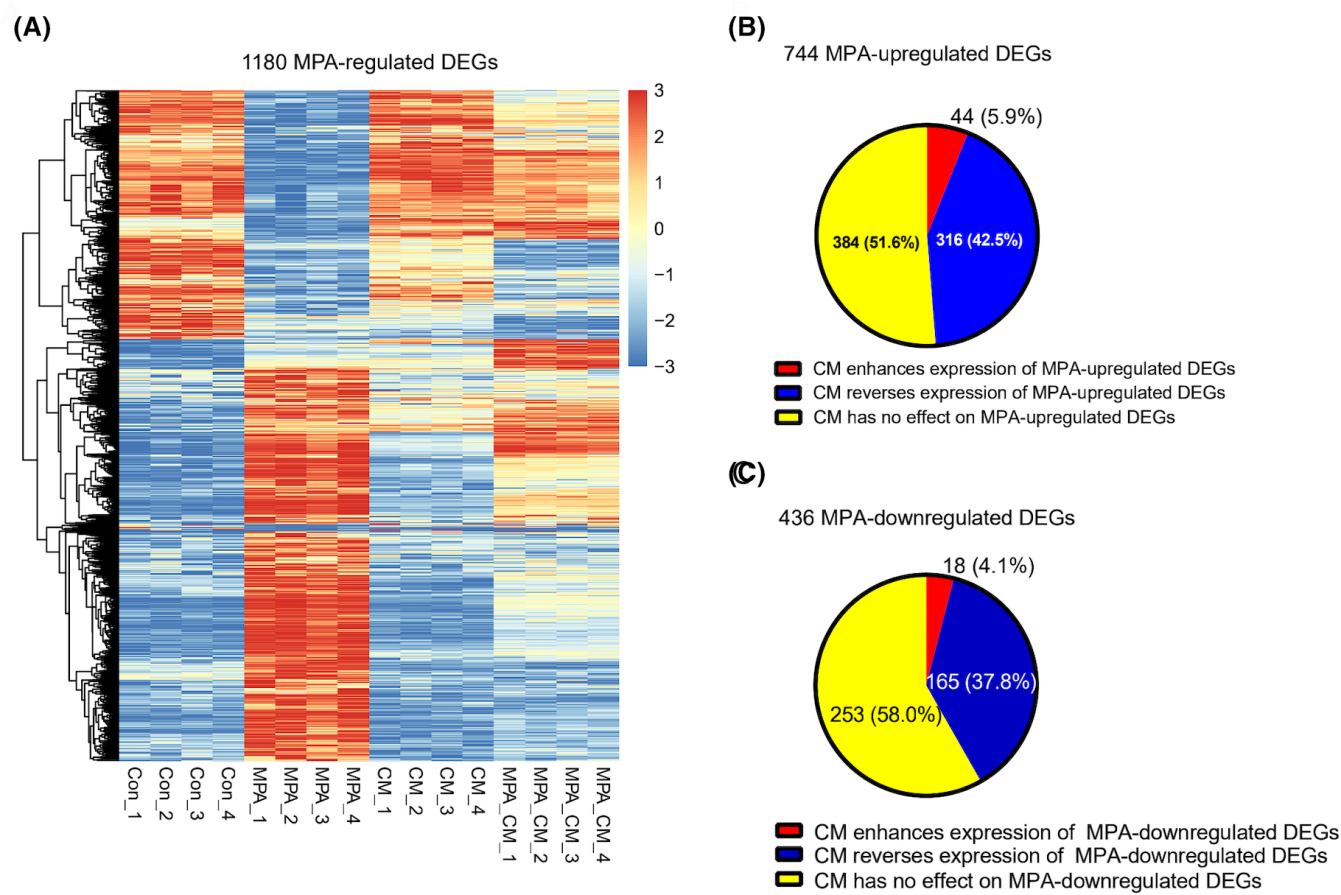
CD163^+^ macrophages antagonize PR signaling and drive EC cell unresponsiveness to progestins. (A) Heat map of 1180 MPA‐upregulated and ‐downregulated DEGs in Con, MPA, CM, and MPA_CM groups. (B) In the MPA_CM group, 316 (42.5%) MPA‐upregulated DEGs were reversed, 384 (51.6%) were unaffected, and only 44 (5.9%) were increased compared with the MPA group. (C) In the MPA_CM group, 165 (37.8%) MPA‐downregulated DEGs were reversed, 253 (58.0%) were unaffected, and only 18 (4.1%) were increased compared with the MPA group.

The data above showed that CD163^+^ macrophages desensitized EC cells to progestins, but the molecular/biological profile changes in EC cells remain unclear. Next, RNA‐seq analysis of Con, MPA, CM, and MPA_CM treatment groups was performed. To confirm the quality of RNA‐seq data, Pearson's correlation was calculated based on the read counts (Figure S4A), and principal component analysis was performed according to the amount of data variability (Figure S4B). Both analyses showed clustered samples by group. The mRNA expression profiles were presented in Figure S4C. The numbers of upregulated and downregulated DEGs were calculated according to the Con or MPA group (Figure S4D). To determine how CD163^+^ macrophage‐derived CM affects MPA‐regulated DEGs, an expression heat map of 1180 MPA‐upregulated and ‐downregulated DEGs in all samples was generated (Figure 4A). Here, the MPA_CM group tended to show decreased MPA‐upregulated DEGs and increased MPA‐downregulated DEGs compared with the MPA group. Among 744 MPA‐upregulated DEGs, the expression of 42.5% was reversed, 51.6% remained unaffected, and only 5.9% were increased in the MPA_CM group (Figure 4B). Similarly, among 436 MPA‐downregulated DEGs, the expression of 37.8% was reversed, 58.0% remained unaffected, and only 4.1% were decreased in the MPA_CM group (Figure 4C). Taken together, we concluded that CM derived from CD163^+^ macrophages was likely to antagonize PR signaling in EC cells by blocking or even reversing the expression of MPA‐regulated DEGs.

### 3.5 CD163 ^+^ macrophage‐induced profile alterations determined by RNA‐seq and ATAC‐seq

**FIGURE 5:**
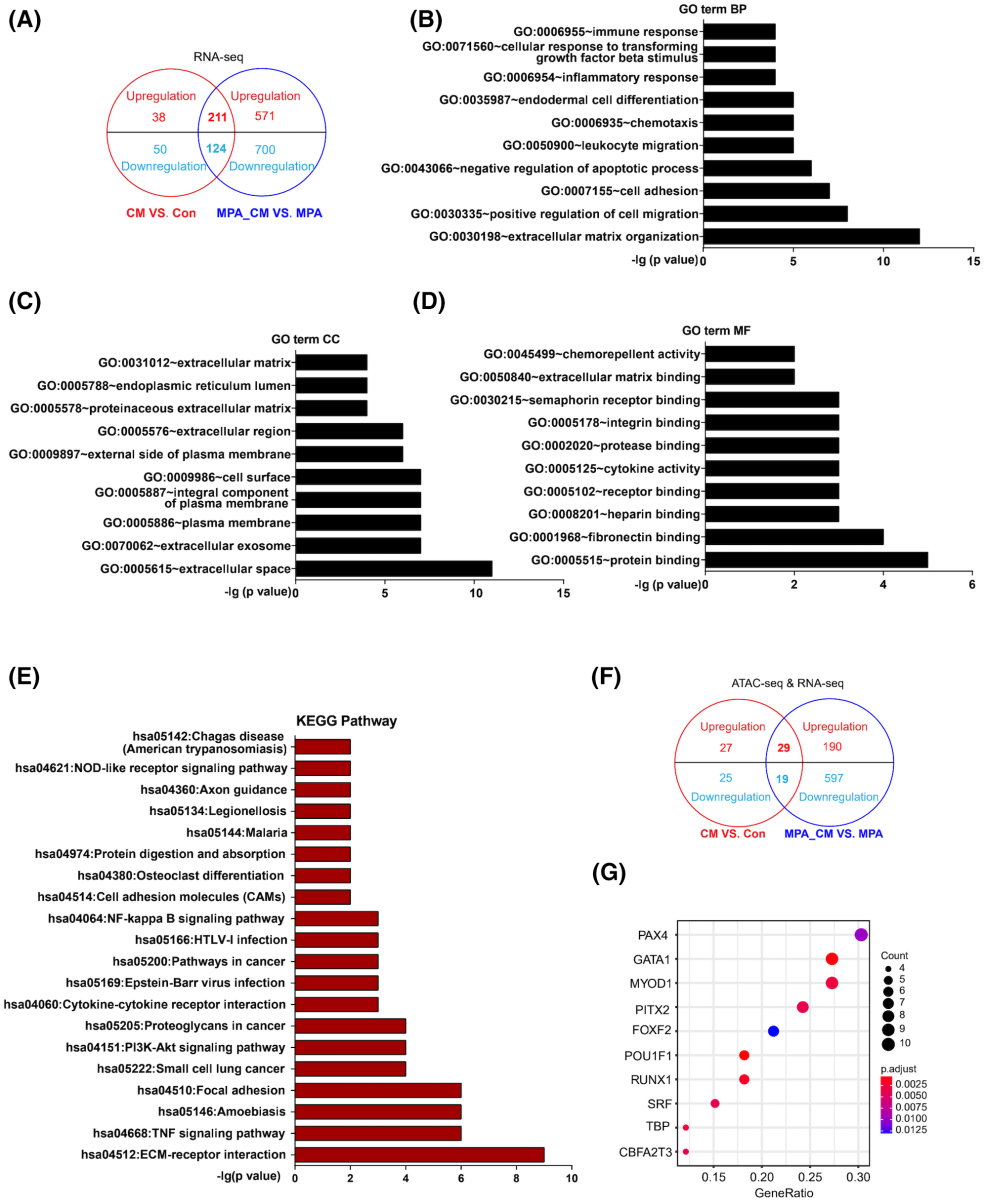
CD163^+^ macrophage‐induced profile alterations determined by RNA‐seq and ATAC‐seq. (A) Overlapping upregulated (211) and downregulated (124) DEGs between CM versus Con and MPA_CM versus MPA from RNAseq analysis. (B–D) GO term analysis of 335 overlapping DEGs and top ten GO terms for BP, CC, and MF. Black represents a significant term with *p* < 0.05. (E) KEGG annotation was generated, and the top twenty pathways were listed. Red represents a significant term with *p* < 0.05. (F) Overlapping upregulated (29) and downregulated (19) DEGs between CM versus Con and MPA_CM versus MPA from the integrated analysis of RNA‐seq and ATAC‐seq. (G) Candidate transcription factors were generated from the overlapping DEGs in (F) using homer peak analysis.

To further explore the mechanism by which CD163^+^ macrophages antagonize PR signaling in EC cells, DEGs from CM versus Con and MPA_CM versus MPA were merged and 211 overlapping upregulated DEGs and 124 overlapping downregulated DEGs were generated (Figure 5A). To understand the functional alterations in the 335 dysregulated DEGs, gene ontology (GO) terms were generated, and the top ten terms were shown in Figure 5B–D. The GO terms for the biological process (BP) category included extracellular matrix organization, positive regulation of cell migration, and cell adhesion (Figure 5B). Similarly, the GO terms for the cellular component (CC) category were significantly enriched in extracellular space, extracellular exosome, plasma membrane, integral component of plasma membrane, cell surface, external side of plasma membrane, extracellular region, proteinaceous extracellular matrix, endoplasmic reticulum lumen, and extracellular matrix (Figure 5C). In addition, the molecular function category included protein binding, fibronectin binding, heparin binding, receptor binding, cytokine activity, protease binding, integrin binding, semaphorin receptor binding, extracellular matrix binding, and chemorepellent activity (Figure 5D). Furthermore, KEGG pathway analysis was performed on these overlapping DEGs, and the enriched pathways included ECM‐receptor interaction, TNF signaling pathway, focal adhesion, PI3K‐AKT signaling pathway, cytokine‐cytokine receptor interaction, pathway in cancer, NF‐kappa B signaling pathway, cell adhesion molecules, and others (Figure 5E). Based on the GO and KEGG analyses, extracellular matrix‐related signaling was significantly enriched in CD163^+^ macrophage‐induced EC cells. Taken together, these findings suggested that extracellular matrix (ECM) related mechanisms might be involved in the CD163^+^ macrophage‐mediated desensitization of EC cells to progestin.

To accurately obtain transcriptional regulatory sequence information based on chromatin accessibility, ATAC‐seq was performed (Figure S5A). The M‐A plot after normalization showed opening and closing peaks in genes relative to the corresponding control (Figure S5B). Then, the opening genes in ATAC‐seq and upregulated DEGs in RNA‐seq were overlapped. Similarly, the closing genes in ATAC‐seq and downregulated DEGs in RNA‐seq were overlapped. The number of overlapped genes was shown in Figure S5C. The targeted DEGs with chromatin open or closed regions were obtained from merged CM versus Con and MPA_CM versus MPA groups based on the integrated analysis of ATAC‐seq and RNA‐seq (Figure 5F). Based on the targeted DEGs, 10 candidate transcription factors, including PAX4, GATA1, MYOD1, PITX2, FOXF2, POU1F1, RUNX1, SRF, TBP, and CBFA2T3, were identified by homer peak analysis (Figure 5F). Within them, FOXF2, POU1F1, and RUNX1 are also involved in regulating ECM‐related signaling, which is consistent with GO and KEGG pathway enrichment analyses. In summary, through RNA and ATAC‐seq analysis, ECM signaling and ECM‐related transcription factors, FOXF2, POU1F1, and RUNX1, were identified to be the critical molecular factors underlying IL10/TGFβ induced progestin desensitization.

## 4 DISCUSSION

In this study, we first found that the increased number of infiltrating CD163^+^ macrophages was significantly associated with progestin insensitivity and prolonged the treatment duration needed to achieve CR in AEH/EEC patients. The CD163^+^ macrophage number was negatively correlated with PR protein expression in human AEH/EEC tissues. Further investigation confirmed that CD163^+^ macrophage‐derived CM and secreted cytokines, including IL10 and TGFβ, decreased the MPA response and PR protein expression level in EC cells. RNA‐seq analysis demonstrated that CD163^+^ macrophages antagonized PR signaling by blocking or reversing the expression of MPA‐regulated DEGs, thereby promoting EC cell unresponsiveness to progestins. Based on GO and KEGG pathway enrichment analyses of DEGs and the integrated analysis of RNA‐seq and ATAC‐seq data, extracellular matrix‐related signaling was identified to potentially be involved in CD163^+^ macrophage‐induced progestin insensitivity in EC cells. Our study identified CD163^+^ macrophages as an important mediator of progestin desensitization and an unfavorable factor for the efficacy of fertility‐preserving treatment in AEH/EEC patients.

Increased infiltrating CD163^+^ macrophages might be a manifestation of chronic inflammation. Recently, clinical trials revealed a strong association between chronic inflammatory status and progestin insensitivity in AEH patients who received progestin‐based fertility‐sparing treatment. Yang et al. reported that obesity and insulin resistance were associated with lower cumulative CR rates and longer treatment times to achieve CR, indicating declined progestin sensitivity in AEH patients.^10^, ^26^ In obese subjects, adipose tissue expansion is associated with increased secretion of several inflammatory cytokines, such as IL6, IL8, and MCP‐1.^27^, ^28^ These inflammatory cytokines, especially MCP‐1, actively recruit circulating monocytes to tissues, which later differentiate into anti‐inflammatory or pro‐inflammatory macrophages.^29^ Moreover, insulin resistance‐related factors regulate the immune microenvironment during the very early stages of endometrial hyperplasic lesions. Therefore, factors derived from obesity‐ or insulin resistance‐induced chronic inflammation might affect the recruitment and polarization of macrophages in AEH/EC patients.

PR status is an important factor that largely reflects the progestin response rate. Factors that interfere with PR signaling, including decreased active PR protein or even loss of PR expression, PR‐related cofactor dysfunctions, or abnormal signaling activation (e.g., TGFɑ/EGF, TGF‐EGFR, PI3K/AKT signaling, and other pathways), desensitize EC cells to progestins. In this study, we found that CD163^+^ macrophages impaired the progestin response in EC cells. Here, we highlight that the number of infiltrating CD163^+^ macrophages was negatively correlated with PR protein in AEH/EC tissues, and CM derived from CD163^+^ macrophages decreased the protein expression of PR. These findings suggested that decreased PR expression was an important event in the microenvironment of CD163^+^ macrophages.

An extracellular matrix‐related mechanism might regulate the progestin response. Based on the results of GO, KEGG, and transcription factor predictions, extracellular matrix‐related signaling was significantly enriched in CD163^+^ macrophage‐induced PIS EC cells. The extracellular matrix is composed of a variety of fibrillar components and non‐fibrillar molecules, which form complex networks that actively communicate with cells through binding to cell surface receptors and/or matrix effectors. Previous studies showed that Ishikawa cells aggregated to form glandular‐appearing tubular and spherical structures when plated on basement membrane extract, and the response of Ishikawa cells to progestin was increased in the presence of CM from stromal cells. Moreover, early decidualization in canines is accompanied by extracellular matrix remodeling in vivo. We postulate that abnormal extracellular matrix remodeling might induce progestin insensitivity, but further studies are needed.

The analysis of chromatin accessibility via ATAC‐seq not only identifies regulatory regions for transcription but also infers transcription factor activity within them. To assess the relationship between chromatin accessibility and gene expression, correlation analysis of peaks at the promoter or distal regions and genome‐wide gene expression in endometrial cancer cells cultured in the presence or absence of CM was conducted (Figure 5G). Among these transcription factor candidates enriched at the regions of altered accessibility, POU1F1 is known to regulate several genes involved in pituitary development and hormone expression. In addition to the well‐known effects on growth hormone and prolactin gene transcription, it has been demonstrated that CD163^+^ macrophages induce POU1F1 expression in breast cancer cells. The overexpression of POU1F1 in cancer cells increases CXCL12 chemokine secretion, which promotes monocyte recruitment to the tumor microenvironment and macrophage transformation into CD163^+^ macrophages^.^ ^30^, ^31^ POU1F1 activation may possibly correlate with cancer cells and M2‐like macrophage polarization and promote CD163^+^ macrophage‐induced progestin insensitivity through a positive feedback mechanism. DEG enrichment emphasized that modulation of the extracellular matrix played an important role in CD163^+^ macrophage‐induced progestin insensitivity in endometrial cancer. Several studies have previously reported that candidate transcription factors, including MYOD1, FOXF2, and RUNX1, transcriptionally regulate the expression of ECM genes and ECM remodeling^,^ ^32^, ^33^, ^34^ suggesting that these transcription factors are potentially involved in progestin insensitivity. Additionally, Bone'y‐Montoya et al. reported that FOXF2 could directly bind to distal *PR* gene regions^,^ ^35^ indicating that FOXF2 may be a key regulator of *PR* gene expression. These effects of candidate transcription factors require further investigations.

The effects of M2‐like macrophages on PR expression seem to be diverse in different tumors. Lindsten et al. demonstrated that CM derived from M2‐like macrophages suppressed expression of estrogen receptor alpha (ERα), but not PR in breast cancer^.^ ^36^ The discrepancy between the study by Lindsten et al. and our findings may be attributed to the heterogeneity of tumor cells and macrophages. Even though breast and endometrial cancer are both hormone‐dependent, emerging evidence supports distinct roles of PR in the pathogenesis of these two cancers. It has been demonstrated that PR signaling has diverse effects on reproductive tissues. In the breast, progestin acts in concert with estrogen to promote proliferative and pro‐survival gene programs. In sharp contrast, progestin inhibits estrogen‐driven growth in endometrium and protects endometrium from neoplastic transformation. Based on the heterogenous nature of PR, the influence of M2‐like macrophages on PR expression may differ between endometrial and breast cancers. Besides, M2‐like macrophages, including M2a, M2b, M2c, and M2d, may also have varied influences on PR expression on breast and endometrial tissues^.^ ^37^ Although these M2 subsets share some markers and immunosuppressive functions, different subsets are induced by different mechanisms and have diverse physiological functions. Recent studies have found significant differences among macrophages from distinct tumors. It has been demonstrated that the percentage of M2c‐like macrophages was significantly higher in advanced (stages II and III) breast cancer^.^ ^38^ However, the predominant M2 subset in hormone receptor‐positive breast cancer and endometrial cancer remain unclear. The detailed landscape of M2‐like macrophages must be deciphered with the integration of new technologies, such as multiplexed immunohistochemistry (mIHC) and single‐cell RNA‐seq (scRNA‐seq), for analyzing which M2‐like macrophage subsets regulated PR signaling in breast and endometrial cancer.

Our study has limitations that should be noted. First, the mechanism underlying decreased PR expression in the CD163^+^ macrophage microenvironment remains unknown. Second, how CD163^+^ macrophages induced progestin sensitivity in EC cells was not definitively answered. Whether extracellular matrix‐related mechanisms are involved in CD163^+^ macrophage‐induced progestin insensitivity needs further investigation.

In summary, our study revealed that a high CD163^+^ macrophage number was significantly associated with progestin insensitivity in AEH/EEC patients. By utilizing in vitro experiments, we suggested that IL10/TGFβ derived from CD163+ macrophages desensitized EC cells to progestin by downregulating PR mRNA and protein expression, and through RNA and ATAC‐seq analysis, ECM signaling and ECM‐related transcription factors, FOXF2, POU1F1, and RUNX1, were identified to be the critical molecular factors underlying IL10/TGFβ induced progestin desensitization. Our study highlighted CD163^+^ macrophages as an important mediator of progestin desensitization and an unfavorable factor for the efficacy of fertility‐preserving treatment in AEH/EEC patients.

## AUTHOR CONTRIBUTIONS

**Lulu Wang:** Conceptualization (equal); data curation (equal); formal analysis (equal); investigation (equal); visualization (equal); writing – original draft (equal). **Qiaoying Lv:** Conceptualization (equal); data curation (equal); formal analysis (equal); writing – review and editing (equal). **Pengfei Wu:** Data curation (equal); methodology (equal); supervision (equal). **Shuhan Luo:** Data curation (equal); validation (equal). **Sijia Liu:** Data curation (equal); supervision (equal). **Xiaojun Chen:** Conceptualization (equal); funding acquisition (equal); supervision (equal); writing – review and editing (equal). **Xuezhen Luo:** Conceptualization (equal); funding acquisition (lead); resources (equal); supervision (equal); writing – review and editing (equal).

## FUNDING INFORMATION

This research was funded by Natural Science Foundation of Shanghai, grant number 18ZR1405300 and Shanghai Medical Centre of Key Programs for Female Reproductive Diseases, grant number 2017ZZ010616.

## CONFLICTS OF INTEREST

No potential conflicts of interest are disclosed.

## Supporting information


Figure S1

Figure S2

Figure S3

Figure S4

Figure S5

Appendix S1

Table S1

## Data Availability

The data that support the findings of this study are available from the corresponding author upon reasonable request.
