# Supplementary figures and images for: RNA‐seq and ATAC‐seq analysis of CD163 + macrophage‐induced progestin‐insensitive endometrial cancer cells
Source: Cancer Med. 2022 Nov 14;12(5):5964–78. doi: 10.1002/cam4.5396 (PMC10028121; doi:10.1002/cam4.5396)

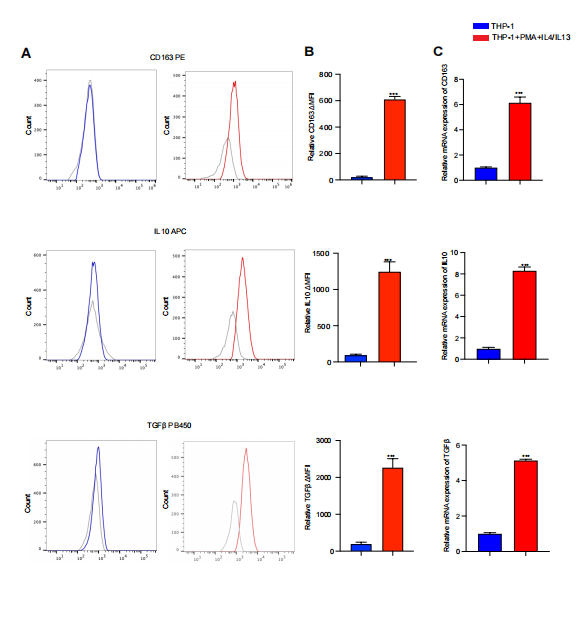

Supplement: Supplementary file 1 — Figure S1 [file CAM4-12-5964-s003.tif]

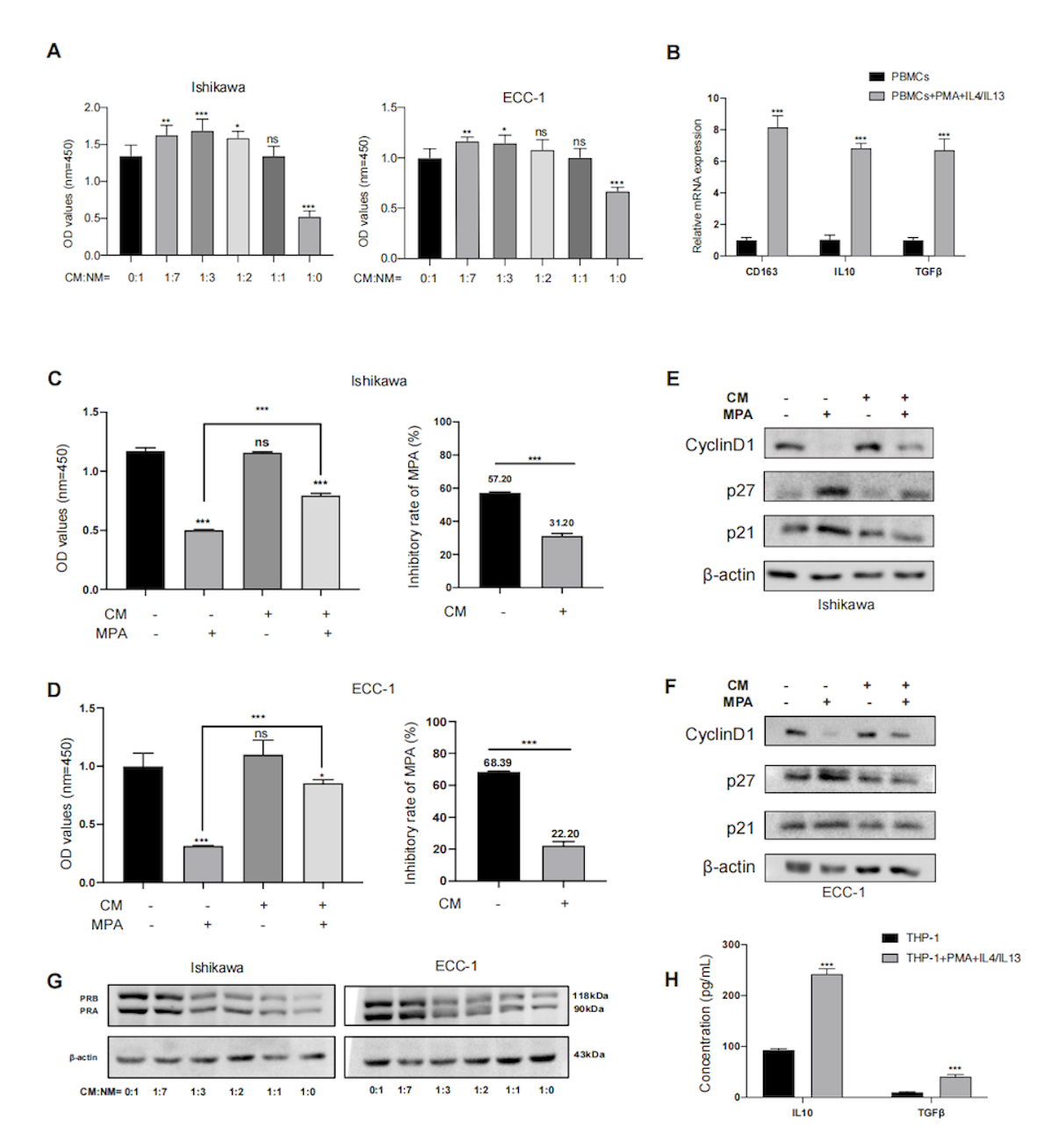

Supplement: Supplementary file 2 — Figure S2 [file CAM4-12-5964-s002.tif]

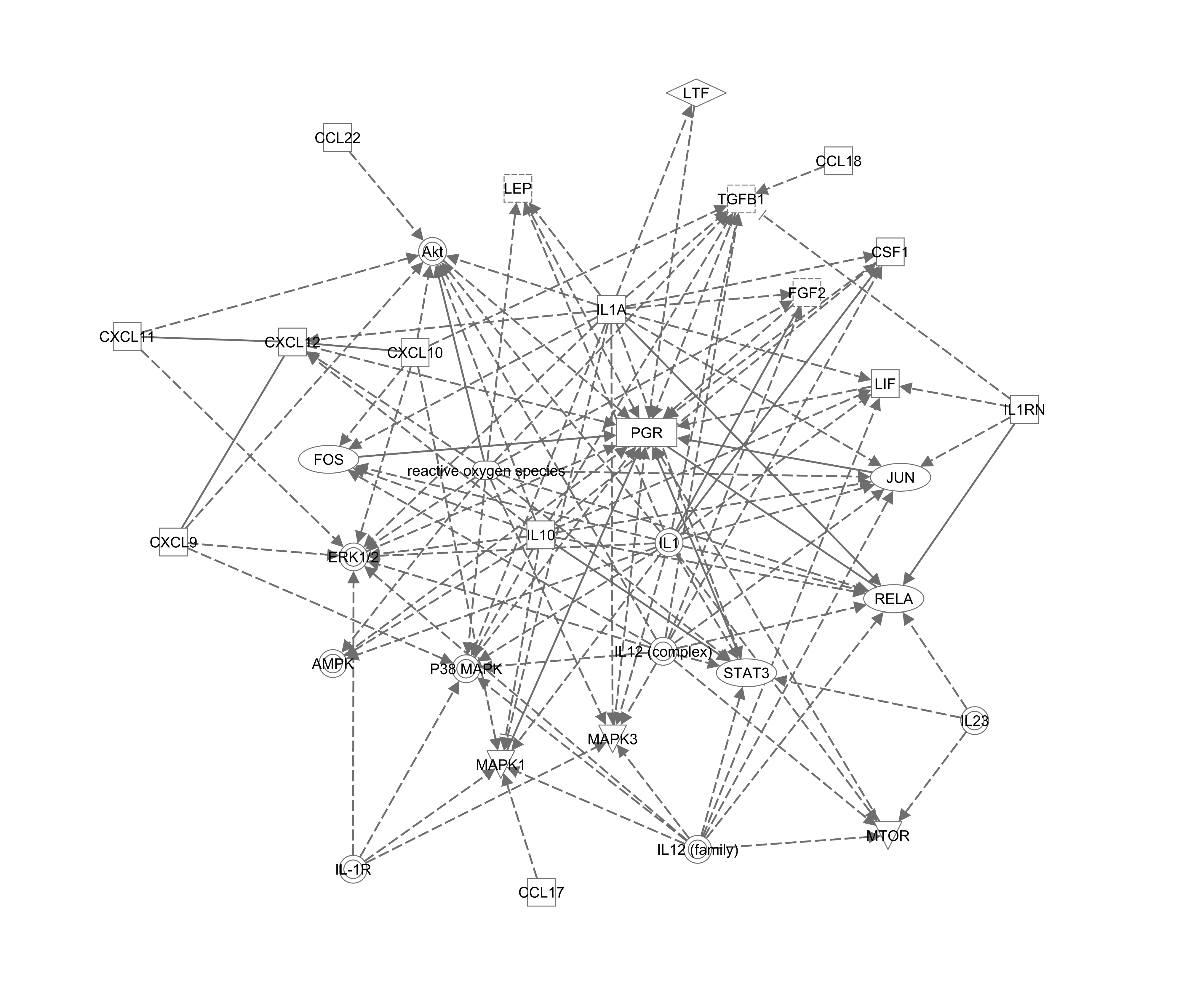

Supplement: Supplementary file 3 — Figure S3 [file CAM4-12-5964-s004.tiff]

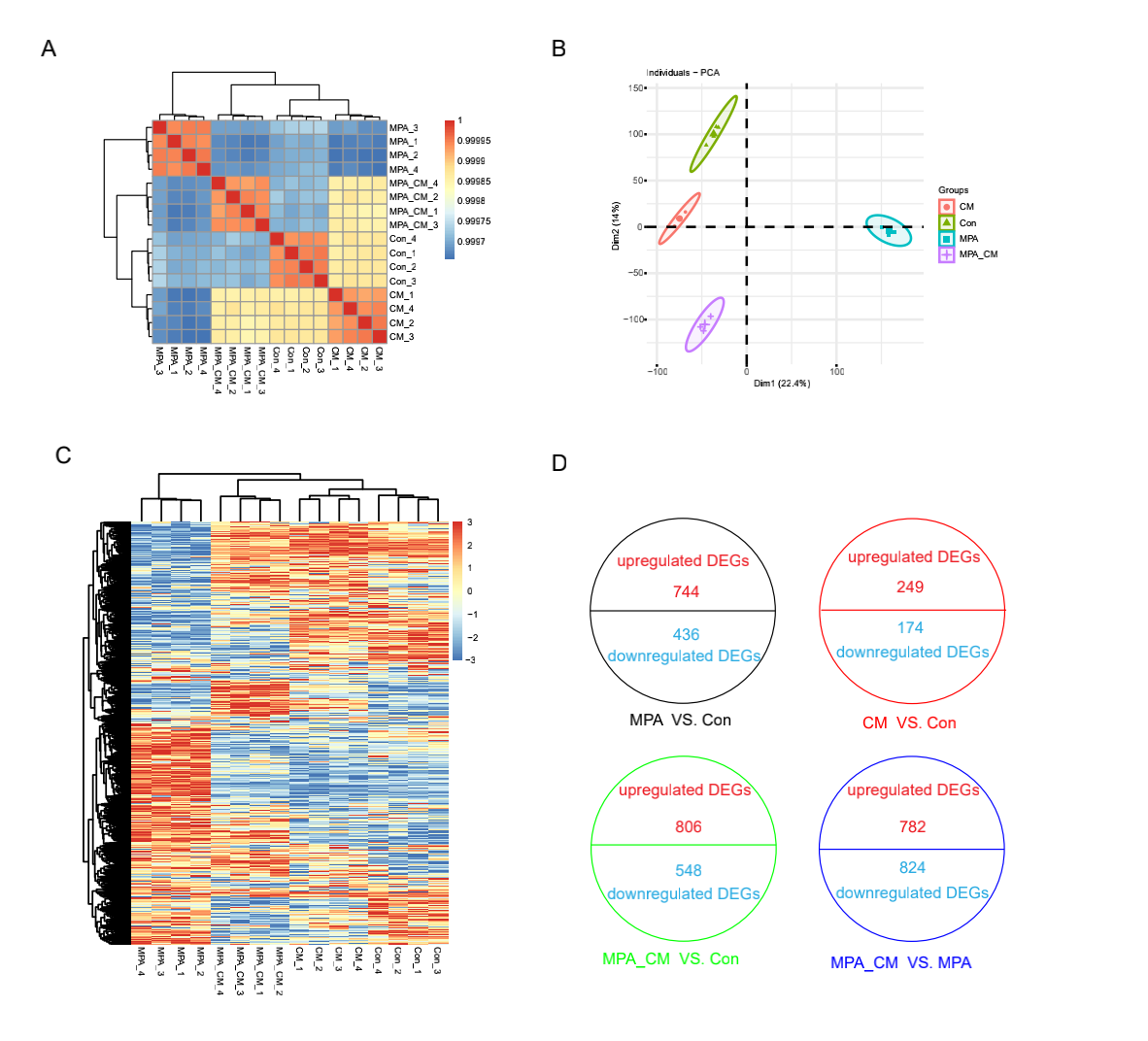

Supplement: Supplementary file 4 — Figure S4 [file CAM4-12-5964-s005.tif]

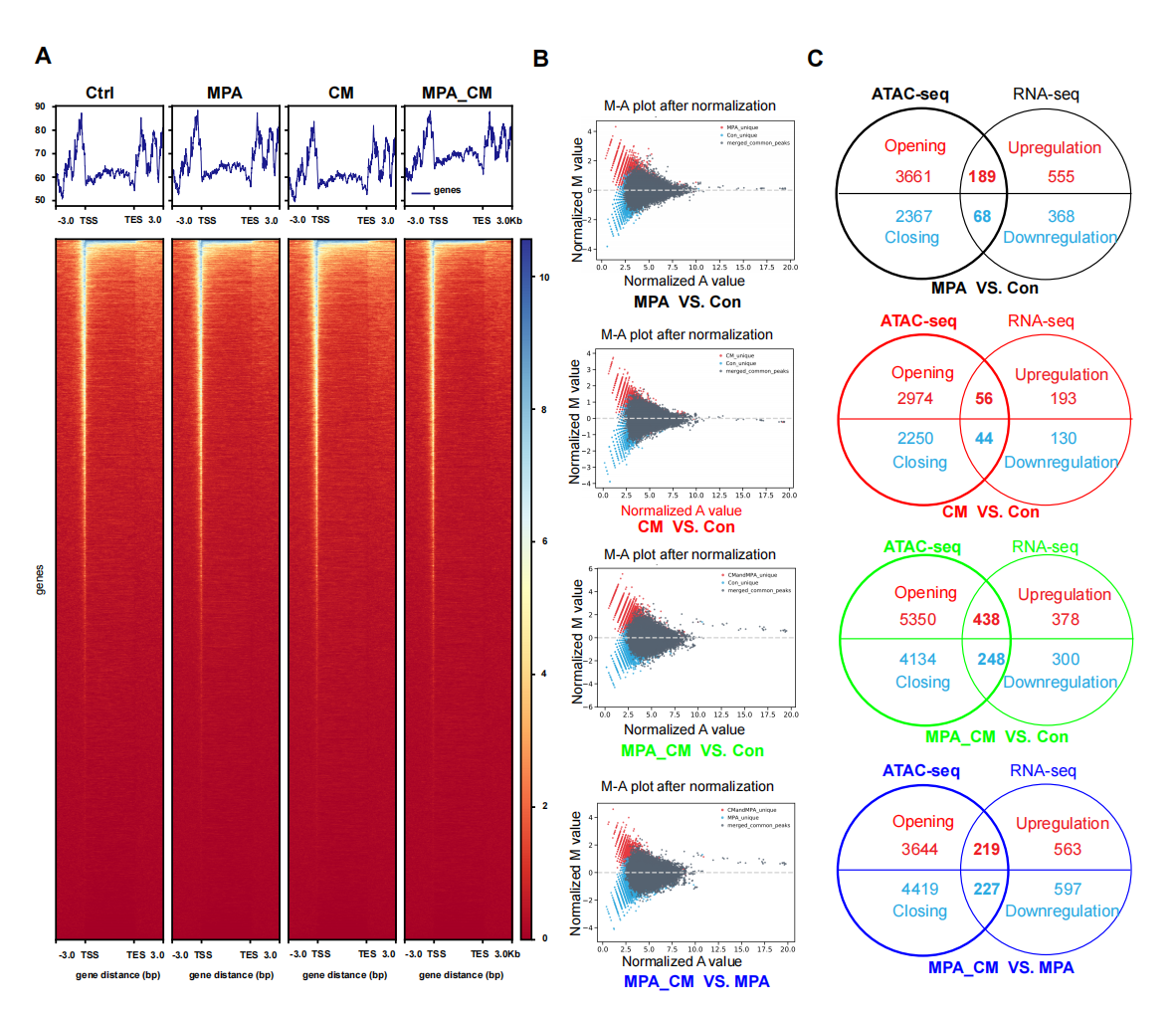

Supplement: Supplementary file 5 — Figure S5 [file CAM4-12-5964-s001.tif]
