# Supplementary material for: RNA‐seq and ATAC‐seq analysis of CD163 + macrophage‐induced progestin‐insensitive endometrial cancer cells
Source: Cancer Med. 2022 Nov 14;12(5):5964–78. doi: 10.1002/cam4.5396 (PMC10028121; doi:10.1002/cam4.5396)
Supplement: Supplementary file 6 — Appendix S1 [file CAM4-12-5964-s006.docx]

### ATAC-seq peak calling

bowtie2 --very-sensitive -X 2000 -x $Bowtie2Index -1 ${sample}*_1.fq.gz -2 ${sample}*_2.fq.gz -p 8 2> $${sample}.bowtie2.log | samtools sort -@ 8 -G 4G -O bam -o ${sample}.sorted.bam

samtools view -h -b -F 1024 ${sample}.sorted.bam > ${sample}.rmDup.bam

samtools index -@ 8 -q 30 $WORKDIR/bowtie2/${sample}.rmDup.bam

macs2 callpeak -f BAMPE -g hs --keep-dup all --cutoff-analysis -n ${sample} -t $WORKDIR/bowtie2/${sample}.rmDup.bam --outdir macs2/${sample} 2> macs2.log

### RNA-seq-based gene expression quantification

STAR --runThreadN 20 --genomeDir genome_index --readFilesIn ${fq1} ${fq2} --outFileNamePrefix ${sample}. --readFilesCommand zcat --outSAMstrandField intronMotif

samtools sort -m 10G -@ 4 -o ${sample}.sort.bam ${sample}.Aligned.out.sam

stringtie -e -B -p 8 -G gene.gtf -o stringtie/${sample}/${sample}.gtf ${sample}.sort.bam
