# Supplementary material for: RNA‐seq and ATAC‐seq analysis of CD163 + macrophage‐induced progestin‐insensitive endometrial cancer cells
Source: Cancer Med. 2022 Nov 14;12(5):5964–78. doi: 10.1002/cam4.5396 (PMC10028121; doi:10.1002/cam4.5396)
Supplement: Supplementary file 7 — Table S1 [file CAM4-12-5964-s007.docx]

| Genes | Primer Sequence (5' to 3') |
| --- | --- |
| PGR | F: ATGGGCACTGTGGAGATAACT |
|  | R: GGCAGGTTTAATCAGAGCAGT |
| CD163 | F: CATCCCGTCAGTCATCCTTTA |
|  | R: GCTGTCTCTGTC TTC GCT TTT |
| IL10 | F: GTTTTCCCTGACCTCCCTCTA |
|  | R: GCTCCCTGGTTTCTCTTCCTA |
| TGFβ | F: ACCTGAACCCGTGTTGCTCT |
|  | R: CGCCAGGAATTGTTGCTGTA |
| GAPDH | F: GGGAAGGTGAAGGTCGGAGTC |
|  | R: AGGGGCCATCCACAGTCTTCT |

Table S1 The sequences of the primers for RT-qPCR
